# Basilar dolichoectasia with intermural hematoma accompanied by cerebral microbleeds and white matter hyperintensities: A case report

**DOI:** 10.1097/MD.0000000000027022

**Published:** 2021-08-20

**Authors:** Sui-yi Xu, Ruo-jun Wang, Lei Zhang, Chang-xin Li

**Affiliations:** a Department of Neurology, The First Hospital of Shanxi Medical University, Taiyuan, China; b Department of Radiology, The First Hospital of Shanxi Medical University, Taiyuan, China.

**Keywords:** basilar dolichoectasia, case report, cerebral microbleeds, intramural hematoma, white matter hyperintensities

## Abstract

**Rationale:**

The clinical manifestations of basilar dolichoectasia (BD) are variable. The diagnosis is based on imaging measurements. Digital subtraction angiography displays only the dilated vascular lumen and lacks visualization of the arterial wall. High-resolution Magnetic resonance imaging (MRI) can identify intramural hematoma; therefore, it may be more suitable for the imaging evaluation of BD. However, most of the existing literature pertaining to BD lacks vascular wall assessment.

**Patient concerns:**

A 65-year-old Chinese man perceived weakness of the left upper and lower limb, double vision, dizziness, nausea, and vomiting was admitted to the emergency department. Fifteen years prior to this admission, he began taking levamlodipine besylate inconsistently for hypertension, but the level of blood pressure control was uncertain. The patient's father had a family history of hypertension.

**Diagnoses:**

An emergency axial computed tomography scan of the brain showed basilar artery (BA) dilation. Computed tomography angiography further indicated a maximum BA diameter of 38.94 mm. The length was >182 mm. MRI revealed acute infarctions of the right medulla oblongata and pons. Meanwhile, the patient had evidence of cerebral small vessel disease, including cerebral microbleeds and white matter hyperintensities. Whole-exome sequencing eliminated significant genetic variations consistent with clinical phenotypes. BD and intramural hematoma were further confirmed by high-resolution MRI of the arterial wall.

**Interventions:**

Atorvastatin was admitted according to the results of the high-resolution MRI of the arterial wall. Benidipine hydrochloride was selected as a long-term anti-hypertensive drug.

**Outcomes:**

The patient had no symptoms of neurological damage during 3-month follow-up.

**Lessons:**

Current evidence shows that BD has no obvious correlation with atherosclerosis. BA dissection and uncontrolled hypertension may be important factors in the progression of BD. BD-related stroke is likely to recur, and there are no standard secondary prevention measures. BD is often accompanied by cerebral microbleeds, and bleeding risk must be assessed during secondary prevention. When the BA diameter is greater than 10 mm, anti-platelet medication should be used with caution, blood pressure should be strictly controlled, and endovascular treatment should be considered.

## 1 Introduction

The clinical manifestations of basilar dolichoectasia (BD) are variable. In addition to causing posterior circulation cerebral infarction and hydrocephalus, BD may cause hemifacial spasm^[1]^ or trigeminal neuralgia^[2]^ due to nerve compression. Mechanical compression by BD may affect the periaqueductal gray of the midbrain, the suprachiasmatic nucleus, hypothalamic-pineal axis function, or melatonin secretion, causing hypnic headache.^[3]^ Morphological analysis of the basilar artery (BA) in the Chinese population showed that the average BA diameter in men was 3.2 ± 0.5 mm and in women was 3 ± 0.5 mm.^[4]^ As early as 1986, Smoker et al^[5]^ suggested that in the mid-pontine BA, a diameter of 4.5 mm should be considered the lower limit of BA dilation. There is also a view that when the BA diameter is greater than 4.5 mm at any point along its path, it can be diagnosed as BA dilation,^[6]^ while a length exceeding 29.5 mm is considered abnormal.^[7]^ Scoring of the degree of BA lengthening based on the height of the BA bifurcation is as follows: score 0, at or below the dorsum sellae level; score 1, in the suprasellar cistern; score 2, between the suprasellar cistern and the third ventricle; score 3, higher than the third ventricle. The deviation scores based on the dorsum sellae or slope are as follows: score 0, median; score 1, paramedian; score 2, between the paramedian and the edges; score 3, beyond the edges or cerebellopontine angle. If BA height score is >2 or deviation score is >2, and the diameter is greater than 4.5 mm, BD can be diagnosed.

Digital subtraction angiography is the reference standard for diagnosing cerebrovascular disease. However, it is rarely used in cases of intracranial arterial dolichoectasia (IADE) because it shows only the dilated vascular lumen and lacks visualization of the arterial wall. With BD, an intramural hematoma is usually located between the media and adventitia, causing arteries to dilate rather than to narrow. Therefore, digital subtraction angiography often fails to show double-lumen signs.^[7]^ The image quality of high-resolution MRI of blood vessel walls is similar to that of digital subtraction angiography, and it can identify an intramural hematoma, which may make it more suitable for the imaging evaluation of BD, and the guidance for the secondary prevention. Unfortunately, most of the existing literature pertaining to BD lacks vascular wall assessment.

This report describes a case of BD with intramural hematoma confirmed by high-resolution MRI of the arterial wall, accompanied by cerebral microbleeds (CMBs) and white matter hyperintensities (WMHs). Whole-exome sequencing eliminated significant genetic variations consistent with clinical phenotypes.

## 2 Case presentation

A 65-year-old man perceived left lower limb weakness when he went to the toilet at 01:00 AM. The patient fell asleep again until 06:00 AM. Upon awakening, the patient had double vision, weakness of the left upper and lower limb, dizziness, nausea, and vomiting. Self-assessed blood pressure was 170/100 mm Hg. The patient was admitted to the emergency department of our hospital at 13:00 PM on the same day. The patient's blood pressure had been found elevated during a physical examination at the age of 18, but this had been dismissed. Fifteen years prior to this admission, he began taking levamlodipine besylate inconsistently for hypertension, but the level of blood pressure control was uncertain. The patient's father had a family history of hypertension.

**Figure 1:**
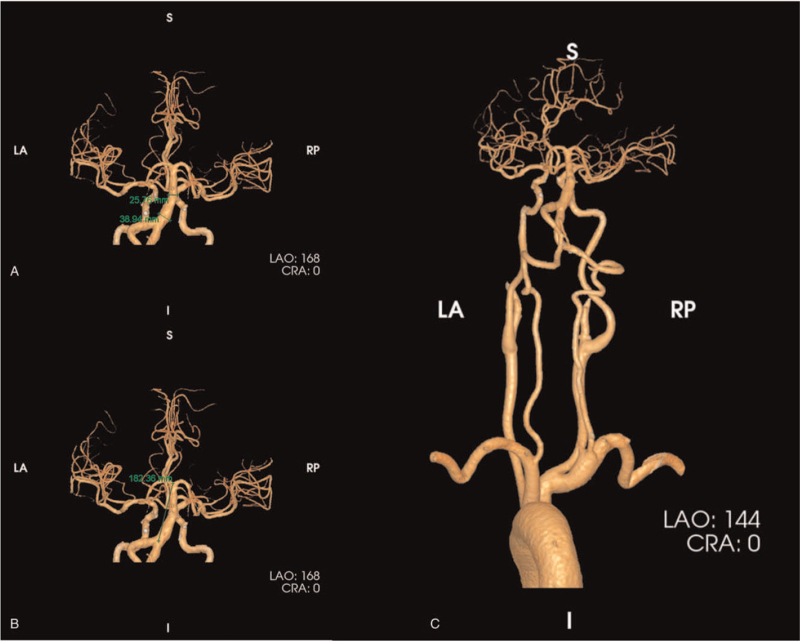
CTA of the intracranial and extracranial arteries. (A) From the 168° LAO perspective, the basilar artery was significantly dilated. Using workstation software, the diameter of the widest portion of the basilar artery was approximately 38.94 mm, and the diameter of the middle portion was approximately 25.76 mm. (B) Due to the tortuosity of the basilar artery, its actual length was greater than 182.36 mm. (C) The remaining intracranial and extracranial arteries were essentially normal. CTA = computed tomography angiography, LA = left anterior, LAO = left anterior oblique view, RP = right posterior.

On admission, the patient had quite evident dysarthria. Eye movements were restricted to the right, and horizontal nystagmus occurred when looking leftward. Muscle strength of the left limb was grade 5-, while the Babinski sign was positive on the left. An emergency axial computed tomography scan of the brain showed BA dilation. Computed tomography angiography further indicated a maximum BA diameter of 38.94 mm (Fig. 1A). The length was >182 mm (Fig. 1B). There was no significant expansion of the remaining intracranial or carotid arteries (Fig. 1C).

The patient's laboratory data, including blood cell counts; serum electrolytes, glucose, glycosylated hemoglobin, and cholesterol; parameters of liver, kidney, and thyroid function; coagulation parameters; levels of D-dimer, cardiac enzymes, myoglobin, and troponin; treponema pallidum antibody; hepatitis antigens and antibodies; human immunodeficiency virus antibody; rheumatism series; and folacin and vitamin B12 levels, were found to be within normal limits. The homocysteine level was slightly elevated.

**Figure 2:**
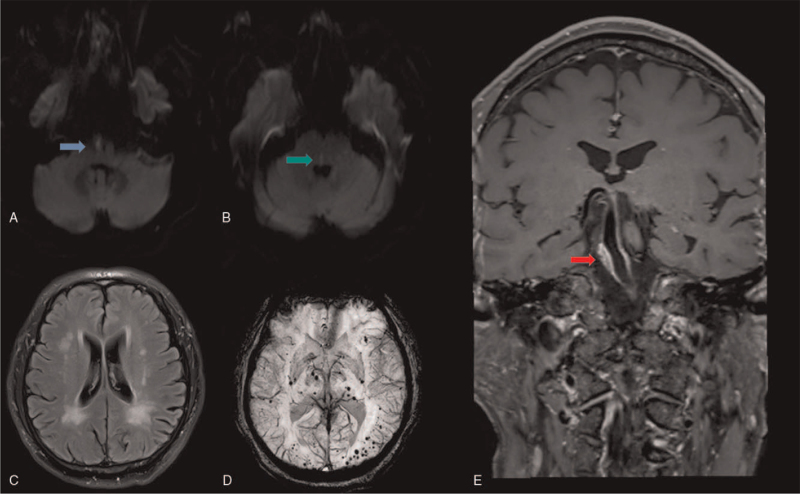
Brain MRI and high-resolution MRI of the arterial wall. (A) DWI suggested high signal in the right medulla oblongata (blue arrow). (B) There was high signal in the right pons (green arrow). (C) FLAIR imaging indicated white matter hyperintensities near the bilateral cerebral ventricles. (D) Cerebral microbleeds on SWI. (E) Localized thickening of the right basilar artery wall with abnormal signals suggesting intramural hematoma on high-resolution MRI (red arrow). DWI = diffusion-weighted imaging, FLAIR = fluid attenuated inversion recovery, SWI = susceptibility weighted imaging.

**Figure 3:**
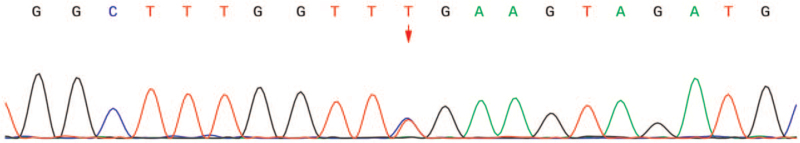
Heterozygous mutation of *GSS* gene (chr20:33516707: C>T).

MRI revealed acute infarctions of the right medulla oblongata (Fig. 2A) and pons (Fig. 2B). WMHs could be seen in the bilateral ventricle and posterior horn of the ventricle (Fig. 2C). Meanwhile, a susceptibility weighted imaging sequence showed CMBs (Fig. 2D). The BA was dilated with a thickened wall, and the vessel wall was more visible on the right than on the left. An intramural BA hematoma was confirmed by high-resolution MRI of the arterial wall (Fig. 2E). Because a gene mutation may be the possible mechanism of BD and some cerebral small vessel diseases (CSVDs), including CMBs, the patient underwent whole-exome sequencing. This identified a heterozygous mutation in the *GSS* gene, but it was not consistent with the clinical phenotype, considering the lack of clinical significance (Fig. 3). Atorvastatin was admitted according to the results of the high-resolution MRI of the arterial wall. Benidipine hydrochloride was selected as a long-term anti-hypertensive drug. The patient was discharged after 2 weeks of hospitalization. The weakness of the left upper and lower limb, double vision, dizziness, nausea, and vomiting disappeared completely. The patient had no symptoms of neurological damage during 3-month follow-up.

## 3 Discussion and conclusion

BD is a form of IADE. Dolichoectatic vessels often have a thin arterial wall, internal elastic lamina degeneration, medial thinning, and smooth muscle atrophy.^[8]^ BD may be related to spontaneous intradural artery dissection.^[6]^ Some studies have observed the whole process of imaging changes from a normal BA to BD and then to aneurysm after more than 10 years.^[9]^ IADE mainly manifests as a scarcity of medial elastic tissue and degeneration of the elastic layer, while intracranial atherosclerosis is a stenotic arterial disease mainly manifesting as lipid infiltration and inflammation of the arterial intima.^[10]^ Interestingly, IADE has no significant correlation with traditional risk factors for atherosclerosis such as age, hypertension, and diabetes.^[11]^ Ultrasound imaging studies have found that there is no correlation between IADE and ultrasound markers of atherosclerosis such as common carotid artery intima-media thickness and plaque.^[12]^ Therefore, for patients with BD, attention should be paid to changes in the arterial wall other than the characteristics of atherosclerotic plaque.^[13]^ Arterial dissection and intramural hematoma may cause a BA hemodynamic disturbance.^[14,15]^ Uncontrolled high blood pressure may be a factor in the acceleration of BD.^[16]^

Pathological observations have found that the risk of IADE in patients with CSVD was 4 times higher than in patients without IADE.^[11]^ CSVD has been defined as the presence of sclerosis and hyalinosis, lipohyalinosis, and isolated fibrinoid necrosis on microscopic examination.^[17]^ The radiological features of CSVD include lacunar infarction, WMHs, enlarged Virchow-Robin spaces, and CMBs.^[18]^ IADE often has combined CSVD features such as WMHs and enlarged Virchow-Robin spaces.^[19]^ Compared to patients without IADE, patients with IADE are more likely to have WMHs in the deep white matter.^[20]^ Studies have reported that BD is related to WMHs and CMBs but is not related to lacunar infarction and enlarged Virchow-Robin spaces.^[21]^ Although the probability of BD combined with CMBs is high,^[22]^ there is no pathological evidence of amyloidosis in patients with IADE.^[11]^

Current studies in molecular biology suggest that IADE and CSVD may be related to abnormal vascular remodeling driven by an abnormal matrix metalloproteinase (MMP)/tissue metalloproteinase inhibitor pathway. Pico et al^[23]^ reported that the MMP-3/5A genotype is related to IADE, suggesting that MMPs play a role in the development of IADE. Recently, Chinese scholars have verified that the imbalance between MMPs and tissue metalloproteinase inhibitors may serve as a potential bridge between BD, lacunar infarction, and WMHs.^[24]^ Compared to patients with normal BA size, the serum level of MMP-9 and the ratio of MMP-9/tissue metalloproteinase inhibitor-1 levels were both significantly higher in vertigo patients with BD and BA elongation.

Japanese scholars reported a case of BD-related cerebral infarction.^[25]^ The patient had 4 time cerebral infarctions within 6 months and was given 2 intravenous alteplase thrombolysis and anti-platelet treatments, but thrombus still appeared on the BA wall. Within 6 months, the diameter of the BA expanded from 15.2 to 24.6 mm. This suggests that BD-related cerebral infarction is not a contraindication to thrombolysis. Early thrombolysis may be effective, but BD-related cerebral infarction is likely to recur.

How do we address the secondary prevention of BD-related cerebral infarction? The pulsating BA can compress the surrounding brain tissues and cause infarction. Uncontrolled hypertension is an important cause of the progression of BD; it is the consensus of the existing literature that blood pressure should be controlled first. Which is more appropriate – anti-platelet or anti-coagulation therapy? As mentioned before, no obvious correlation exists between BD-related cerebral infarction and atherosclerosis. BD may cause perforating arteries to be twisted, blocked, and thrombosed in situ.^[8]^ If a BA hemodynamic disturbance is found (such as blood flow stasis), anti-coagulation may be suitable. If evidence of arterial-arterial embolism caused by unstable plaque is found, anti-platelet and statin therapy may be more appropriate. It is worth noting that BD is often accompanied by CMBs, and bleeding risk must be assessed during secondary prevention. When the BA diameter is greater than 10 mm, anti-platelet medication should be used with caution, blood pressure should be strictly controlled,^[26]^ and endovascular treatment should be considered.^[10]^ Of note, BD may be part of a systemic arterial disease accompanied by coronary artery dilation and abdominal aortic aneurysm.^[13]^ It will be valuable to assess the entire arterial tree in future clinical practice.^[19]^

## Acknowledgments

We would like to thank Editage (www.editage.com) for English language editing.

## Author contributions

**Conceptualization:** Ruo-jun Wang.

**Data curation:** Ruo-jun Wang, Lei Zhang.

**Project administration:** Sui-yi Xu, Ruo-jun Wang.

**Resources:** Sui-yi Xu.

**Software:** Sui-yi Xu, Lei Zhang.

**Supervision:** Chang-xin Li.

**Writing – original draft:** Sui-yi Xu.

**Writing – review & editing:** Chang-xin Li.
